# Widespread supplementary feeding in domestic gardens explains the return of reintroduced Red Kites *Milvus milvus* to an urban area

**DOI:** 10.1111/ibi.12237

**Published:** 2015-01-28

**Authors:** Melanie E Orros, Mark D E Fellowes

**Affiliations:** People and Wildlife Research Group, School of Biological Sciences, University of ReadingReading, RG6 6AS, UK

**Keywords:** anthropogenic feeding, habitat associations, raptor, reintroduction, urban ecology

## Abstract

Reintroductions are commonly used to mitigate biodiversity loss. One prominent example is that of the Red Kite *Milvus milvus*, a charismatic raptor of conservation concern. This species has been reintroduced across the UK over the last 25 years following its near extinction after centuries of persecution. The species was not expected to recolonize urban areas; its historical association with human settlements is attributed to scavenging on human waste and refuse, a resource now greatly reduced on the streets of modern European cities. However, the species has become a common daytime visitor to a large conurbation centred on the town of Reading, southern England, approximately 20 km from the first English reintroduction site. Given a near-absence of breeding and roost sites, we investigated foraging opportunities and habitat associations that might explain use by Red Kites of this urban area. Surveys of discarded human foods and road-kill suggested that these could support at most 13–29 Kites per day. Face-to-face surveys of a cross-section of residents revealed that 4.5% (equivalent to 4349 households) provided supplementary food for Red Kites in their gardens. Using estimates of per-household resource provision from another study, we calculated that this is potentially sufficient to feed 142–320 Kites, a substantial proportion of the total estimated to visit the conurbation each day (between 140 and 440). Road transects found positive associations between Red Kites and residential areas. We suggest that the decision made by thousands of householders to provide supplementary food for Red Kites in their gardens is the primary factor explaining their daytime abundance in this urban area.

Reintroductions or translocations of species are increasingly used to combat biodiversity loss (IUCN/SSC 2013). Hundreds have now taken place worldwide, with mixed success (Seddon *et al*. 2007). The UK's largest reintroduction attempt to date is that of the Red Kite *Milvus milvus* (Evans *et al*. 1999, Carter 2007). The species was extirpated from England and Scotland by the late 19th century following sustained persecution, leaving in the UK only a small population persisting in Wales in suboptimal habitat (Lovegrove 1990, 2007). Since 1989, a staged programme has taken place across the UK that, together with recovery of the Welsh population, has increased the UK population from possibly just one successfully breeding female (May *et al*. 1993) to over 2715 breeding pairs by 2013 (Welsh Kite Trust 2013). The conservation significance of the increasing UK population is underlined by the species' continued decline over much of its core European range (Carter 2007).

Prior to their decline in the UK, Red Kites were commonly associated with urban areas, their scavenging diets resulting in royal protection for consumption of anthropogenic waste on the streets of London and other settlements in the Middle Ages (O'Connor 2000, Lovegrove 2007). However, it was thought unlikely that reintroduced Red Kites would also use urban areas to any great extent (Carter 2007) because modern sanitation and refuse management offer fewer foraging opportunities. However, the species has recently become a common daytime visitor to a conurbation centred on the town of Reading (‘Greater Reading’), approximately 20 km south of the first English reintroduction site in the Chiltern Hills. This is despite there being a maximum of just three confirmed breeding sites in Reading and its direct surroundings (Bucknell *et al*. 2013), and no substantial roost sites (N. Bucknell pers. comm. 2013).

Given the almost complete lack of breeding and roosting sites in the area, we assessed what foraging opportunities might be responsible for the common daytime presence of Red Kites in this urban area, where the availability of natural food is relatively scarce and human disturbance and threats are potentially high. The Red Kite is a facultative scavenger with a broad and plastic diet (Cramp & Simmons 1980, Davis & Davis 1981) and is known to exploit anthropogenic foodstuffs (Wildman *et al*. 1998, Carter 2007). Potential ‘inadvertent’ urban food resources include carrion (e.g. road-kill) and discarded human foods (e.g. meat products and bread). Deliberate feeding is another possibility. Although no formal feeding stations exist in the region, villagers in the Chiltern Hills have provided food in their gardens for Red Kites since soon after the reintroduction (Carter & Whitlow 2005) and such feeding is now known to occur across the Chilterns and surrounding regions (Orros & Fellowes 2014). Such provisioning may have diverse effects (both positive and negative), ranging from changes in fitness parameters such as body condition and individual survival probability to increased intraguild competition with other local facultative scavengers (e.g. corvids).

The aims of this study were to investigate the sources and extent of potential foods for Red Kites and the relative numbers of Kites potentially supported by these resources, and the species' daytime habitat associations. We predicted that if the provision of food in gardens is the primary source of food, Red Kites would be more commonly seen over residential areas than other habitat types. If other food sources (e.g. road-kill and discarded human foods) predominate, we predicted that no such association with residential areas would be apparent. In the absence of Kite census data specific to Greater Reading, we also estimated the number of Red Kites using the conurbation daily.

## Methods

### Study area

The study took place in a 72-km^2^ urbanized area in Berkshire, southern England, consisting of the town of Reading (51°27′N, 0°58′W) and the contiguous parishes of Woodley, Earley, Tilehurst, Holybrook and Purley-on-Thames and the electoral ward of Shinfield North. This area covers 96 004 households (Office for National Statistics 2013) and is hereafter referred to as Greater Reading.

### Potential foraging opportunities

#### Food on roads

To estimate the potential food resources available to Red Kites on roads in the study area, two surveyors walked 23.8 km of major roads, comprising four transects from the centre of Greater Reading to the outskirts. Eleven repeats were performed in August–September 2011, with start and end points varied and a minimum 2-day gap between surveys. The survey months reflected surveyor availability but are also likely to represent the peak season for road-kill, as these months directly follow the peak spring breeding season of birds and mammals in the UK. Our observations suggested no obvious seasonal differences in the levels of human waste on the survey routes. Surveyors assigned all potential food observed to a food-type category: road-kill, human foodstuffs (type noted) and rodents/birds from other sources. An approximate mass range was also estimated for each item: ≤ 50, > 50–100 and > 100–150 g for foodstuffs (selected following a pilot study). Carcass masses were estimated by completeness and reference to Harris and Yalden (2008) for mammals or Cramp and Brooks (1985) for birds. We surveyed only major roads (UK category ‘A’) due to their higher traffic flow and therefore probable higher levels of road-kill and waste than minor roads (Department for Transport 2012).

We estimated the median daily mass of potential food available (*M*_road_) for Kites on Greater Reading's major roads (75.8 km A-roads; arcgis) from the per-km median of all transects, using category midpoints for foodstuffs. We acknowledge that this method assumes both daily replenishment and that all food recorded was available to Red Kites. These are unrealistic assumptions, and so the estimate of food availability is a maximum. For example, attractiveness is likely to vary with item type and size, other locally common species such as Eurasian Magpies *Pica pica* and Red Foxes *Vulpes vulpes* also take discarded food and road-kill (Cramp *et al*. 1994, Harris & Yalden 2008), and town-centre streets are cleaned daily (http://www.reading.gov.uk/residents/streetcare-and-environmental-cleansing/).

#### Garden feeding

In a broader questionnaire survey designed to examine awareness of and attitude towards Red Kites, we included a question asking whether respondents fed Red Kites in their garden. We selected 10 supermarkets across Greater Reading and varied times and days of survey sessions to cover a range of different socioeconomic and lifestyle groups. Interview length was no more than 5 min to encourage participation, and interviewers recruited without revealing the subject of the interview in order to avoid bias (Salant & Dillman 1994). Although we cannot guarantee that the respondents in each survey period represent a random sample of all those shopping within that time-frame, everyone exiting the shop during the survey session was approached by one of two surveyors unless both were already conducting interviews. We conducted two rounds of interviews, in November 2010–January 2011 and July–September 2011, with one respondent per household and no repeat participation. For cases in which postcodes were identical (UK postcodes may cover up to approximately 80 households) and the answers were similar, we used only the first respondent as a conservative measure to guard against pseudoreplication.

We compared the socioeconomic profile of our sample with that of southeast England using the CACI (2010) A Classification Of Residential Neighbourhoods (ACORN) UK geodemographic system. This allocates households to categories using their postcode and is based on census data (see Supporting Information Table S1 for brief category descriptions and CACI 2010 for further details). Following data collection, we found that the proportions of Greater Reading respondents in three ACORN categories were similar to southeast England (‘wealthy achievers’, ‘comfortably off’ and ‘hard-pressed’: differences = 0.00–0.01), but one (‘urban prosperity’) was over-represented (0.16 vs. 0.10) and another (‘moderate means’) under-represented (0.08 vs. 0.13), with a *G*-test indicating significant variation overall (*G *= 61.525; *P *< 0.0001). However, Reading (65% of Greater Reading households) has more ‘urban prosperity’ (0.31) and slightly fewer ‘moderate means’ (0.11) residents than southeast England and so, given also the precautions against bias described above, for the present purposes we considered the sample representative of the study area.

### Numbers of Kites potentially supported by foraging opportunities

We calculated the number of Red Kites that could potentially be supported by food on roads in Greater Reading each day (*K*_road_) separately for minimum and maximum food requirement estimates for the species (*F*_daymin_ = 80 g; *F*_daymax_ = 180 g; Carter 2007) by:


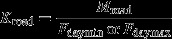


where *M*_road_ is the daily median mass (in g) of potential food on roads in Greater Reading.

The equivalent measure for the Kites potentially supported daily by garden feeding in Greater Reading (*K*_garden_) was:


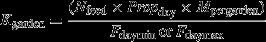


where *N*_feed_ is the total households feeding in Greater Reading (from the face-to-face surveys); *Prop*_day_ is the proportion of feeding households putting out food on any given day, estimated at 0.28 (Orros & Fellowes 2014); and *M*_pergarden_ is the estimated median mass believed by householders to be taken by Kites per garden per day, estimated at 21 g (Orros & Fellowes 2014).

We note that *K*_road_ and *K*_garden_ are derived from estimates, each of which has its own 95% confidence interval (95%CI) or interquartile range, and thus should be regarded as estimates themselves. To avoid compounding errors, we present the ranges as the values obtained using *F*_daymin_ and *F*_daymax_.

### Daytime habitat associations of Kites

Two transects, bisecting Greater Reading as close to north–south (10.2 km) and east–west (15.1 km) as possible given the road layout, were driven between 11:00 and 15:00 h GMT in late January–mid-March 2011 and 2012. A pilot study indicated more sightings of Kites during these hours and we selected the season to maximize visibility (most trees along the routes are deciduous). Twelve east–west and 10 north–south repeat surveys were carried out in 2011, and six of each in 2012. The day of the week was varied to minimize bias but road layout prevented reversal of the east–west route and so both were driven in one direction (north to south and east to west). Observers differed between years.

A car with front-seat observer travelled at 25 mph subject to traffic. For each sighting of a Kite within a transect of 100 m width, centred on the road midpoint, we recorded: position along the route to 0.1 mile (from mileometer); the number of Kites seen; their position relative to the road (left/right/above); sighting band (0–10 m from road midpoint (directly over road/pavement); 10–30 m either side of road midpoint (approximately first row of buildings or equivalent in open/industrial areas); 30–50 m (approximately gardens/second row of buildings)). The widths of the sighting bands were selected following pilot studies with range-finders, and measurement in Environmental Systems Research Institute (ESRI) arcgis v. 10. Surveyors also recorded Red Kites seen beyond the outer sighting bands for reference. As 100% detection of Red Kites within the transect was assumed (see below), we did not consider the distance from the road of each sighting band in our analyses. Instead, the bands were used as a means of isolating each sighting to a smaller area than the full width of the transect.

We mapped the routes and sighting bands onto an Ordnance Survey map. We added habitat data from EDINA Digimap (http://digimap.edina.ac.uk/) within arcgis and then calculated the areas of three habitat categories: ‘residential’ (houses, flats, gardens), ‘natural’ (open/wooded, e.g. parks) and ‘other’ within the transect area. We selected ‘residential’ and ‘natural’ because of garden feeding and use by Kites of open areas, respectively (Cramp & Simmons 1980). Roads, pavements and paths were manually assigned to adjoining categories, and were split along their midpoints when habitats differed on opposite sides.

We then split the transects into 160-m sections (equivalent to the 0.1-mile distance recordings from the vehicle mileometer). Each Red Kite sighting was then assigned to the appropriate section and sighting band. The boundaries of these subsections created ‘sighting’ polygons whose areas were unequal as the driven routes were not straight. We allocated each Red Kite sighting to the dominant habitat by area in its polygon. Because we aimed to investigate habitat associations rather than absolute numbers and are unable to exclude the possibility that some Kites seen on different survey dates may have been repeat sightings of the same birds, we used the total number of sightings across repeat visits as a measure of Kite activity in each habitat type rather than of the number of birds seen.

### Estimated number of Red Kites visiting Greater Reading during daytime

To estimate how many Red Kites visit Greater Reading each day, we considered the relevant information obtained in the different elements of this study. From the investigation of foraging opportunities, *K*_garden_ (as calculated above) was used as an approximate measure of Kite numbers (see Results for why *K*_road_ was not considered here). From the habitat association work, we estimated Red Kite numbers by calculating the overall means of the mean densities of Kites in residential, natural and other habitats (sightings divided by area of habitat) per repeat visit for the N–S and E–W transects. We then multiplied these approximate densities by the respective areas of each of these habitats within Greater Reading (31.1, 31.1 and 9.8 km^2^, respectively; Generalised Land-Use Database 2005), equating residential habitat with ‘domestic buildings’ + ‘domestic gardens’, natural habitat with ‘greenspace’, and other habitats with ‘non-domestic buildings’ + ‘water’ + ‘other land uses’. We note that transects were driven when activity was greatest (M. Orros unpubl. data), and clustering of birds was sometimes apparent. Furthermore, our broad habitat categories make it likely that densities varied within them (by e.g. housing type). We also acknowledge the value of detection curves to account for declines in detection rates with increasing distance from transect routes in density estimation (Bibby *et al*. 2000). However, previous raptor surveys have assumed 100% detection (e.g. Millsap & Lefranc 1988) and we considered it a reasonable approximation of relative density here given our narrow transects, the generally sparse vegetation on our survey routes and that Kites were in flight and could often be seen beyond transects (recorded by surveyors; data not shown).

### Statistical analysis

All calculations required to estimate *K*_garden_ and *K*_road_ were carried out in excel (Microsoft Inc.). To examine habitat associations, we compared the observed Kite activity within habitats with expected values from within-transect habitat proportions using *G*-tests of association in excel or Fisher's exact tests in r 2.120 (R Development Core Team 2010) when values were < 5 (Crawley 2007). We analysed years separately to account for annual variation and different observers. Significance was accepted at *P* ≤ 0.05 and two-tailed tests were used throughout.

## Results

### Potential foraging opportunities

#### Food on roads

Estimated road-food masses/km/day varied by type (Table1). *M*_road_ was estimated at 2300 g. Road-kill constituted approximately 41% of the total road food by mass but few items (16 in total). Non-road-kill carcasses were one Wood Mouse *Apodemus sylvaticus* and one Common Blackbird *Turdus merula*.

**Table 1:** Estimated mean masses and summary statistics per km per day of the potential foods available to Red Kites on 23.8 km of Greater Reading's major roads recorded on walked transects in August–September 2010 (11 repeats). Human foods were assigned to mass categories (based on pilot study; range midpoints used for estimates): ≤ 50, > 50–100 and > 100–150 g

Food type	Mass/km major road/day (g)
Road-kill	16.0
Other vertebrate carcasses	0.4
Discarded human foods	
Meat-based	4.6
Not meat-based	18.6
Mean of all food types	40
Median of all food types	31
Q1–Q3	16.7–62.5
Range	0–131.8

#### Garden feeding

We obtained 949 (503 first repeat, 446 second) responses from the face-to-face surveys that were from postcodes within our definition of Greater Reading. This corresponds to survey coverage of 1.0% of households. 4.53% (95%CI: 3.30–6.06) of respondents fed Red Kites (non-significant difference between rounds: *P *= 0.805). *N*_feed_ was therefore estimated at 4349 (95%CI: 3168–5818).

#### Numbers of Kites potentially supported by foraging opportunities

The number of Kites potentially supported daily by food on major roads (*K*_road_) is 13–29 and the number by garden feeding (*K*_garden_) 142–320, using *F*_daymax_ and *F*_daymin_ for the lower and upper values of each range, respectively.

### Daytime habitat associations of Kites

Significant associations existed for the E–W transect in both years and for N–S in 2012 but not 2011, with a higher level of Kite activity than expected observed over residential areas and lower than expected levels over natural and other habitats (Table2). Fisher's exact test was also significant for E–W in 2012 (*P* < 0.0001).

**Table 2:** Observed (O) and expected (E) Red Kite activity according to habitat proportions from driven transect surveys of Greater Reading with associations tested using *G*-tests (values given in parentheses after *P*-values in table where appropriate) or Fisher's exact tests as appropriate. Habitat definitions: residential (houses, flats, gardens); natural (open/wooded land, e.g. parks); other. Roads, pavements and paths were assigned to adjoining categories, split along the midpoint when habitats differed on opposite sides. Kite activity was measured as the total number of sightings across repeats per habitat type, as the possibility of repeat sightings across repeats could not be excluded. Kite sightings were allocated to 160-m sections along routes and to a sighting band (0: 0–10 m either side of road midpoint; 1: 10–30 m either side of road midpoint; 2: 30–50 m either side of road midpoint). The most common habitat within the polygon created by section and sighting-band boundaries was used in analyses. Repeat numbers: 2011: 12 E–W, 10 N–S; 2012: both 6

Route	Habitat	Within-transect habitat proportion	Kite activity				*P* (*G*)	
			2011		2012		2011	2012
			O	E	O	E		
North–south	Residential	0.544	19	17	34	23	*P *=* *0.827	*P *=* *0.016
	Natural	0.086	3	3	1	4		
	Other	0.370	10	12	8	16		
East–west	Residential	0.593	132	103	86	57	*P *< 0.001 (22.214)	*P *< 0.001 (41.301)
	Natural	0.185	20	32	5	18		
	Other	0.222	22	39	6	22		

### Estimated number of Red Kites visiting Greater Reading during daytime

From our investigation of foraging opportunities, we considered only *K*_garden_ (142–320) in the estimation of Kite numbers because this is based on food that garden-holders believed Kites had taken (*M*_pergarden_ from Orros & Fellowes 2014). By contrast, *K*_road_ is based solely on food seen on roads.

From our habitat association data, the estimated mean densities of Kites were 10 (N–S = 6; E–W = 14), 3 (N–S = 2; E–W = 4) and 4 (N–S = 3; E–W = 5) individuals/km^2^ for the residential, natural and other habitats, respectively. Multiplying by the respective areas of each of these within Greater Reading gives an estimated total of 444 (range: 278–609 using highest and lowest means given above) individuals across the study area.

Taking the lower and upper values from the above estimates, we suggest that approximately 140–440 Kites visit Greater Reading daily, rounding to the nearest 10 individuals.

## Discussion

Our results suggest that Red Kites forage over the Greater Reading urban area during daylight hours mainly because householders feed them in their gardens. Red Kite activity observed during driven transects was generally significantly higher in residential areas than in other habitats. Furthermore, on any one day, enough food to support up to 320 individuals is believed to be taken by Red Kites from gardens within Greater Reading. This is an order of magnitude more than potential food availability on major roads. This amount of food could theoretically support most of the Red Kites that we estimate visit Greater Reading each day (*c*.* *140–440). Although, as noted, our estimate of Kite numbers is only an approximation, for comparison there are estimated to be more than 1000 breeding pairs in the wider Chilterns region (Welsh Kite Trust 2013). The actual number of individual Kites making some use of these foraging resources may be greater given that complete reliance on garden feeding or food found on roads by individual Kites is unlikely (Elliott *et al*. 2006, Jones & Reynolds 2008, Orros & Fellowes 2014).

The relatively low abundance of potential food for Red Kites observed on roads contrasts with the assumed importance of road-kill in the diet of reintroduced Kites elsewhere in the UK (Carter & Grice 2002). In addition to our survey results, over 3 years, no fieldworker observed or heard of Red Kites foraging from streets or pavements within the study area. This may relate to low speed limits in this urban area reducing the availability of road-kill.

The widespread feeding of Red Kites in gardens in Greater Reading is particularly intriguing because the activity has recently become controversial. Two authors of Red Kite feeding guidance for members of the public (Anon 2006) have now withdrawn their support for feeding, whereas other experts do not view it as problematic if the guidance is followed (e.g. Carter 2007, see Orros & Fellowes 2014 for further details). Carter (2007) identifies the main potential problems as (1) the low nutritional value of cooked foods relative to natural resources, (2) the fact that processed meats contain potentially harmful additives such as salt and (3) absence of skin or bone from food given in gardens potentially causing calcium deficiency, which has been linked to growth and bone disorders in some juvenile Kites. The concerns involving the types of food provided in gardens are consistent with the ‘junk-food’ hypothesis more commonly proposed for seabirds, under which the low value of a supplementary foodstuff decreases breeding success as it is either insufficient or unsuitable for provisioning young, as Grémillet *et al*. (2008) found for Cape Gannets *Morus capensis* feeding on fishery waste. In the context of our study, however, it should be noted that a recent survey of Kite feeding in and around our study area found that most householders provided at least some raw meat that contained skin and/or bone and few gave processed foods (Orros & Fellowes 2014). However, whatever the quality of the supplementary resources provided, the widespread supplementary feeding of Red Kites documented may still have both ecological and evolutionary influences, particularly if continued in the long term. These range from the potential release of poor-quality individuals from selective pressures (e.g. García-Heras *et al*. 2013) to the potential for Red Kites to become overabundant ‘native invaders’ *sensu* Carey *et al*. (2012), as have some other facultative scavengers such as Red Foxes and Brown Rats *Rattus norvegicus*, with consequent effects on other species, for example through intraguild competition.

Although determination of roost and breeding locations was beyond the scope of this study, our work provides an indication that many of the Red Kites visiting Greater Reading must travel relatively long distances from such sites to forage in the area. The Greater Reading conurbation is approximately 9 km north–south and 15 km east–west, with no more than three confirmed breeding sites and no known major roosts, suggesting that most individuals travel from outside the conurbation to forage within it. By contrast, Red Kites in the Midlands of central England typically forage < 3 km from nests (although up to 6 km recorded) and close to winter ranges (Carter & Grice 2002), with work in Wales and Germany recording most within 2–4 km of nests (although up to 20 km known; Cramp & Simmons 1980, Carter 2007). We speculate that predictable provisioning across gardens within Greater Reading may have led at least in part to greater than average foraging distances. Studies involving tracked individuals are required to investigate this further.

We are unaware of other examples of widespread deliberate provisioning of raptors by domestic householders, although other meat-feeding birds are fed in Australia (Jones & Reynolds 2008). However, some raptor species are fed as part of conservation programmes (e.g. Gilbert *et al*. 2007, Gustin *et al*. 2009, Cortés-Avizanda *et al*. 2010). Cortés-Avizanda *et al*. (2010) investigated the use of supplementary feeding stations by six avian scavengers in Spain, including Red and Black Kites *Milvus migrans*, following the drastic reduction in accessible ungulate carcasses because of disease control regulations. These authors recommended the use of many small feeding stations over fewer large ones in order to mimic more closely the spatiotemporal pattern of distribution of single carcasses. This has parallels in the scattered nature of garden feeding. Although the primary focus of these authors was to avoid resource monopolization by larger, more dominant species, this may also apply among age classes within a species. Unintentional anthropogenic provisioning of raptors also occurs, such as from landfill sites (e.g. Blanco 1997, Elliott *et al*. 2006, De Giacomo & Guerrieri 2008) or exposed carcasses (e.g. Shultz *et al*. 2004, Margalida & Colomer 2012), but the importance of these resources to individual survival has received little attention (Oro *et al*. 2013). However, variation in the use of supplementary foods by individual raptors has been documented in Egyptian Vultures *Neophron percnopterus*, in which individuals in poorer body condition made greater use of supplementary food than those in better condition (García-Heras *et al*. 2013). We are unable to test this for our work in the absence of individually marked birds but, if this were the case, this would lend support to the possible release of these individuals from selection pressures present in the absence of supplementary food resources.

Our results are relevant to other reintroductions of Red Kites within or close to urban areas. For example, the release of Red Kites in northeast England began in 2004 in a semi-urban area close to a large conurbation (Northern Kites 2009). Characterization of distributions and supplementary feeding over time and comparison of these areas may reveal whether our findings are a special case or more generally applicable. Monitoring other large conurbations near Reading where Red Kite sightings are currently relatively less common (e.g. Oxford) would also aid investigation of this. In this context, the feeding of other garden birds in Greater Reading is similar to that in five UK cities, including Oxford (Davies *et al*. 2012; Orros & Fellowes in press), a hint perhaps that other urban dwellers might also broaden their bird-feeding habits should the opportunity arise. In this context, our findings are relevant to the planning or monitoring of reintroductions or invasions around urban settlements of any species that might use anthropogenic resources. Individual decisions to feed Kites by thousands of people without involvement in the reintroduction, rather than inadvertent provisioning via road-kill or waste, appear to have collectively influenced the local abundance and distribution of this charismatic species in little over a decade.

We thank K. Grainger, F. Coulson, E. Day, C. Whitelock, N. Mann, T. Hudson, A. Nicholson, L. Daniells and M. Schenk for various fieldwork contributions. We are grateful to all survey participants for their time and to the supermarkets and local councils for granting us permission to conduct the surveys. We also wish to thank the journal editors and anonymous reviewers for their helpful comments and suggestions; these greatly improved the final manuscript. Funding: BBSRC doctoral training award to M.O. Requests for data should be made to the corresponding author.

## Supporting Information

Table S1 Brief description of the aspects of the CACI ACORN geodemographic categories relevant to the present work (see CACI (2010) for full category descriptions).
