# Supplementary material for: Widespread supplementary feeding in domestic gardens explains the return of reintroduced Red Kites Milvus milvus to an urban area
Source: Ibis (Lond 1859). 2015 Jan 28;157(2):230–8. doi: 10.1111/ibi.12237 (PMC4409027; doi:10.1111/ibi.12237)
Supplement: Table S1 — Brief description of the aspects of the CACI ACORN geodemographic categories relevant to the present work (see CACI (2010) for full category descriptions). [file ibi0157-0230-sd1.docx]

# Supporting Information

**Table S1.** Brief description of the aspects of the CACI ACORN geodemographic categories relevant to the present work [see CACI (2010) for full category descriptions]. Categories based on UK 2001 census data and various lifestyle surveys.

| **ACORN category** | **Description** |
| --- | --- |
| Wealthy achievers | Affluent, with incomes well above national average. Typically middle-aged or retired but also families with children. Usually large, detached houses. |
| Urban prosperity | Broad range of incomes; typically above national average. Wide age range (includes students and recent graduates as well as senior managers). Wealthier, older members typically in large houses; younger/less affluent in flats or shared student houses. |
| Comfortably off | Broad category including a range of ages. Usually semi-detached or detached houses. |
| Moderate means | Average to below average incomes. Most often families with children at home. Typically terraced housing. |
| Hard-pressed | Group with lowest income. Broad age range. Usually terraced or semidetached housing on estates. |
